# On the Use of Belladonna, in Arresting the Secretion of Milk

**Published:** 1857-09

**Authors:** J. H. Connally

**Affiliations:** Barnesville, Georgia


### ARTICLE V.

*On the use of Belladonna, in arresting the secretion of milk.* By J. H. CONNALLY, M. D. Barnesville, Georgia, April 10th, 1857.

ALLOW me, through the columns of your Journal, to relate the following case illustrative of the effect of Belladonna, in arresting the secretion of milk. I noticed in one of the back numbers of the Southern Medical and Surgical Journal, where two cases had been treated successfully with that Medicine.

When first it met my eye, I did not think much upon the subject; but since then I have had occasion to try the remedy in the following case: I was called a few days since, to a servant belonging to Mrs. ——, who had been delivered of twins, some days previous to that time. The "little ones" lived only a few moments after birth. On examination of the mother, I found the breasts very much enlarged, knotty and quite tender; pulse full and bounding; stomach nauseated, with considerable headache. I gave her some Medicine to tranquilize the action of the heart, and then applied the breast pump; but it was so very painful, and the lactiferous ducts were so completely obs ructed with what the patient termed "curdled milk," that I was compelled to abandon it. I then ordered v. grains of Belladonna to be dissolved in ℥i of warm water, and applied to the areola. This was done twice during the night, with considerable relief to the patient.

On my visit, the next evening, I found that the tumefication, with all unpleasant symptoms, had entirely disappeared, and the patient declared herself quite well. I did not perceive any ill effects from the use of this medicine. The lochia flowed regularly on, and ceased at the regular time, with subsidence of all unhealthy action. If this proves as effectual in the future, as it has in some few cases that have been related, it is, certainly, a great discovery, and worthy the notice of every Physician.

